# Increasing volume of food by incorporating air reduces energy intake*

**DOI:** 10.1017/jns.2014.43

**Published:** 2014-12-08

**Authors:** Samuel Serisier, Anthony Pizzagalli, Lucie Leclerc, Alexandre Feugier, Patrick Nguyen, Vincent Biourge, Alexander J. German

**Affiliations:** 1 Royal Canin Research Center, Aimargues, France; 2 Nutrition and Endocrinology Unit, Oniris, National College of Veterinary Medicine, Food science and Engineering, France; 3 Department of Obesity and Endocrinology, Institute of Ageing and Chronic Disease, University of Liverpool, Liverpool, UK

**Keywords:** Weight management, Satiety, Volume of food, Canine nutrition, BW, body weight

## Abstract

Major challenges with weight management using weight-loss diets include hunger and rapid consumption of food, both of which lead to poor owner compliance. The aim of the present study was to determine the effect of increasing volume, by incorporating air, into dry expanded food, on satiety in dogs. Three studies have been performed. The first study aimed to measure the effect of volume of food on meal duration in dogs fed at their maintenance energy requirement. The purpose of the second study was to determine the effect of volume of food on satiety. The aim of the third study was to compare the satiety effect of the test diet with a maintenance dry diet commonly used in adult dogs. Throughout the studies, faecal score remained optimal. As volume of diet increased, the duration of food intake significantly increased (*P* < 0·001) and energy intake significantly decreased (*P* = 0·012). The present study has demonstrated that incorporating air into food to increase the volume of diet induces a satiety effect, independent of macronutrient profile, possibly by slowing food intake. Consequently, incorporating air into food might be a useful strategy for weight management in companion animals.

Overweight and obesity are common in dogs and cats, and predispose them to a variety of diseases and decreased longevity^(^^1^^)^. Weight-loss programmes are successful in experimental trials^(^^2^^,^^3^^)^, but do not perform as well in practice^(^^4^^,^^5^^)^. A major hurdle is that energy restriction causes hunger, which leads to increased begging behaviour. This puts increased strain on the owner–animal bond, which can affect owner compliance with the weight-loss programme. Developing strategies to improve satiety would greatly assist in case management.

Evidence from human studies suggests that some foods may be more effective than others in reducing hunger, probably because of the influence of macronutrients in the diet^(^^6^^)^. In pets, foods with increased fibre^(^^7^^)^ or water^(^^8^^)^ content are the most satiating. However, both have limitations; excessive dietary fibre can adversely affect digestibility^(^^9^^)^, while water can affect faecal consistency especially in breeds with lower digestive tolerance^(^^10^^)^.

Increasing meal volume can also increase satiety, and thereby decrease energy intake, and strategies used successfully in human subjects include incorporating air into liquid yogurt^(^^11^^)^ and snacks^(^^12^^)^. However, such an approach has not hitherto been used in companion animals. Therefore, the aim of the present study was to determine the effect of incorporating air into dry expanded food on *ad libitum* food intake in dogs.

## Materials and methods

### Ethical approval

Housing and management protocols adhered to European regulatory rules for animal welfare, while all experimental protocols complied with European Union guidelines on animal welfare and were approved by the Royal Canin Committee for Animal Ethics and Welfare.

### Diets

The test diet was a complete, dry, expanded diet, designed to fulfil maintenance energy requirements in dogs (Table 1). The density of this diet was low (125 g/l) compared with standard maintenance dry diets used in adult dogs (350–400 g/l). For study 2, the satiety effect of the test diet was compared with a control diet with exactly the same formula but a greater density (290 g/l, 4396 kJ/l; Table 1). In the study 3, the satiety effect of the test diet was compared with a maintenance dry diet commonly used in adult dogs (400 g/l, 6479 kJ/l; Table 1)

**Table 1.** Dietary composition of the three diets used in the study

	Test diet		Control diet		Standard maintenance diet*	
ME† content	15 162 kJ/kg (3624 kcal/kg)		15 162 kJ/kg (3624 kcal/kg)		16 297 kJ/kg (3895 kcal/kg)	
Energy density	1895 kJ/l (453 kcal/l)		4397 kJ/l (1051 kcal/l)		6485 kJ/l (1550 kcal/l)	
Mass density	125 g/l		290 g/l		400 g/l	
	ME (g/1000 kcal)	ME (g/MJ)	ME (g/1000 kcal)	ME (g/MJ)	ME (g/1000 kcal)	ME (g/MJ)
Moisture	22	5	22	5	23	6
Crude protein	55	13	55	13	68	16
Crude fat	24	6	24	6	37	9
Crude fibre	6	2	6	2	3	1
Total dietary fibre	24	6	24	6	17	4
Ash	12	3	12	3	15	4
Nitrogen-free extract	156	37	156	37	110	26

*Medium adult dry, Royal Canin, contained dehydrated poultry protein, maize starch, maize, wheat starch, animal fats, dehydrated pork protein, wheat, hydrolysed animal proteins, beet pulp, fish oil, soya oil, yeasts, minerals, hydrolysed yeast (source of manno-oligo-saccharides), trace elements, vitamins and antioxidants. Test diet contained rice, maize starch, wheat, maize gluten, poultry meal, animal fat, hydrolysed soya protein isolate, vegetable fibres, trace elements and vitamins.

^†^ME, metabolisable energy calculated according to NRC2006 equation^(^^13^^)^.

### Study 1: effect of food volume on meal duration

The aim of this study was to determine the effect of altering meal volume, without changing meal energy intake, on meal duration in dogs fed at maintenance energy requirement, and to determine whether differences existed among breeds. Fifteen adult neutered female dogs of various breeds (e.g. Labrador retriever, Fox terrier, Beagle, Golden retriever, Dachshund, Brittany spaniel, German Shepherd dog and Cocker spaniel) were used. The median age was 11·2 (3·2–13·3) years, median body weight (BW) was 17·7 (7·1–42·8) kg and all dogs were in ideal body condition (i.e. body condition score 5/9). The energy requirement to maintain optimal BW had been empirically determined for each dog prior to the study, and the group median was 431 (280–769)  kJ/kg^0·75^/d. To avoid weight gain during the study, the maximum food fed to each dog was its individual daily energy requirement. Daily requirements were divided into two meals of equal size. In order to create four diets with different volumetric energy densities, the dogs' usual diet and the test diet were mixed in different proportions; the final energy densities of the resulting diets were 3344 kJ/l (diet 1), 2855 kJ/l (diet 2), 2370 kJ/l (diet 3) and 1881 kJ/l (diet 4), respectively. Dogs were successively fed the four diets at their individual maintenance energy requirement (in two meals per d) for five consecutive days, with the order of diets determined by Latin square. Energy intake, duration of meals and BW, were recorded as well as faecal score according to the five-point scale previously described^(^^14^^)^. Energy intake was measured by weighing the bowl before and after a meal to determine the amount of food eaten. The energy consumed was then calculated by multiplying the energy content of the food by the amount consumed.

### Study 2: satiety effect of food volume

The aim of this study was to determine the effect of altering volumetric energy density while maintaining macronutrient profile, on satiety in dogs. Ten adult beagle dogs (three intact males, seven neutered females) were used. Median age was 5·6 (2·8–8·2) years, median BW was 11·82 (7·96–14·02) kg, and all were in ideal body condition (5/9) or slightly overweight (6/9). Two diets, with the same macronutrient profile but different volume, were compared using a cross-over design to assess the effect of volume on energy intake. The test diet was compared with an identical diet with higher density (290 g/l, 15 162 kJ/kg, 4397 kJ/l; Table 1). Food intake was measured when diets were fed at hourly intervals, as previously described ^(^^7^^)^. Briefly, individual dogs were offered 502 kJ/kg^0·75^ for 15 min at 08:30 (first meal) and at 09:30 (second meal) and then offered 1005 kJ/kg^0·75^ for 30 min at both 10:30 (third meal) and 11:45 (fourth meal). Each diet was tested three times on three non-consecutive days, and the energy intake at each meal recorded.

### Study 3: satiety effect of test diet compared with standard adult canine maintenance diet

The aim of this study was to compare the satiety effect of the test diet compared with a maintenance dry diet commonly used in adult dogs and which density was higher than the control diet used in the study 2. The test diet (125 g/l, 15162 kJ/kg, 1895 kJ/l) was compared with a standard adult canine maintenance dry diet (Medium adult dry, Royal Canin; 400 g/l, 16 297 kJ/kg, 6485 kJ/l; Table 1) using a cross-over design. Ten adult beagle dogs (two intact males, eight neutered females) were used. Median age was 5·7 (5·0–11·6) years, median BW was 10·4 (8·8–15·9) kg, and all were in ideal body condition (5/9) or slightly overweight (6/9). Four meals were offered at hourly intervals, in the same manner as for study 2, and the energy intake at each meal was recorded by weighing bowl before and after meals.

### Statistical analysis

All data were analysed using the Statistical Analysis Systems Institute package (SAS version 8; SAS Institute Inc.), and the level of statistical significance set at *P* < 0·05, for two-sided analyses. Data were analysed by two-way non-parametric or parametric (as appropriate) ANOVA using the mixed procedure of SAS. Diet, week and their interaction were included as fixed effects and the dog was included as a random term. Results were expressed as median (range) or means with their standard errors as appropriate.

## Results

### Study 1: effect of food volume on meal duration

When dogs were fed their normal diet, median meal duration determined over five consecutive days was 85 (29–430) s. There was no effect of diet on energy intake (*P* = 0·513) but, as greater proportions of the test diet were fed, meal duration increased significantly (*P* < 0·001). In this respect, food intake for diets 1, 2, 3 and 4 was 431 (281–775), 423 (226–762), 427 (272–775) and 423 (285–695) kJ/kg^0·75^, respectively, while meal duration was 171 (55–900), 185 (77–900), 268 (80–900) and 374 (136–900) s, respectively. BW did not change during the course of the study (−3 (−10 to +7) %; *P* = 0·111), faecal score remained optimal throughout, and there were no differences on the different diets.

### Study 2: satiety effect of food volume

None of the dogs ate all of the food offered during the study. Energy intake was lower by 19 (−40 to +4) % with the test diet compared with control diet (*P* = 0·012) (Table 2). This effect remained whatever the week of the test (*P* = 0·605), and there was no diet–week interaction (*P* = 0·438). BW of dogs did not change significantly during the 2 weeks of the study (−0·7 % (−4·3 to +4·1 %), *P* = 0·614).

**Table 2.** Energy intake in kJ/kg^0·75^ for each meal and the total energy intake for the study 2 and study 3

Study 2										
	Test diet					Control diet				
First meal	Second meal	Third meal	Fourth meal	Total	First meal	Second meal	Third meal	Fourth meal	Total
Mean	307**	62**	127	115	611*	400	139	106	108	753
sem	39	17	14	13	42	33	14	16	20	54
Study 3										
	Test diet					Standard maintenance diet				
First meal	Second meal	Third meal	Fourth meal	Total	First meal	Second meal	Third meal	Fourth meal	Total
Mean	445	204***	219*	147	1015***	502	450	346	162	1460
sem	19	28	30	23	67	0	25	69	14	89

*P < 0·05; **P < 0·01; ***P < 0·001 *v.* corresponding meal of control diet for study 2 and standard maintenance diet for study 3.

Study 2: total energy intake was lower with the test diet compared to control diet (*P* = 0·012). This effect remained whatever the week of the test (*P* = 0·605), and there was no diet–week interaction (*P* = 0·438). Study 3: energy intake was lower with the test diet compared with the standard canine maintenance dry diet (*P* < 0·001). This effect remained whatever the week of the test (*P* = 0·214), and there was no diet–week interaction (*P* = 0·472).

### Study 3: satiety effect of test diet compared with a standard adult canine maintenance diet

None of the dogs ate all of the food offered. Over the course of the study, a decrease in energy intake was noted with all diets, and this started at the second meal (*P* < 0·001); energy intake was lower by 31 (−17 to −41) % with the test diet compared with the standard canine maintenance dry diet (Table 2). This effect remained whatever the week of the test (*P* = 0·214), and there was no diet–week interaction (*P* = 0·472). BW of dogs increased significantly when they fed *ad libitum* the standard maintenance dry diet during 1 week (5 (−2 to 7) %; *P* = 0·004), while their BW did not change when they fed *ad libitum* the test diet during 1 week (0 (−9 to 3) %; *P* = 0·418). Faecal score remained optimal throughout the study, whatever the diet.

## Discussion

The present study has demonstrated that using air to increase the volume of dry dog food decreases energy intake and increases meal duration in *ad-libitum*-fed dog. These results confirm and extend findings from previous studies in human subjects demonstrating that using air to increase food volume and decrease energy density, can improve satiety^(^^11^^,^^12^^)^. Other strategies for decreasing the mass:energy ratio of a diet include adding water or dietary fibre, and both are also known to enhance satiety^(^^7^^,^^8^^)^. However, negative effects can be seen with both strategies, including the possibility of soft faeces when using wet food, most notably in breeds at risk of poor stool quality such as German Shorthair Pointers and German Shepherd dogs^(^^10^^)^. In contrast, faecal quality was not affected when dogs consumed the volume-expanded dry diet of the present study.

The exact reason for this effect is not clear. One possibility is that the increase in meal volume results in a longer meal duration, allowing a greater time for the release and effect of gastrointestinal satiety hormones. In previous studies, in obese adolescents, retraining to slow food intake had a profound effect on release of satiety hormones including a decrease in secretion of the orexigenic hormone ghrelin, and an increase in secretion of peptide YY which can decrease appetite^(^^15^^)^. The present study did not aim to determine the physiological basis for such changes, and further studies, for instance measuring ‘satiety hormones’ would now be required to produce a better understanding.

The present studies were designed to assess effects on food intake when the energy balance of dogs was either neutral (study 1) or positive (study 2 and 3) energy balance. The beneficial effect seen on satiety could be of use as a strategy for prevention of weight gain in dogs. Moreover, incorporating air into pet food, to increase volume of food, could have a beneficial psychological impact on owners without providing more energy to companion animals. Indeed, many owners use food as a primary means of showing love to their dogs, and readily feed more when signs of hunger are evident^(^^16^^)^. A diet that enables owners to feed more food, volumewise, without feeding more energy would, thus, be of great benefit. Nonetheless, given that the studies performed were only short-term in nature, it is possible that the effect would not be maintained over time, and further studies would be required to examine the duration of effect. In addition to preventing weight gain, the strategy could help with compliance of obese dogs on weight management regimes. Inadequate compliance of owners is a major problem in the prevention and treatment of pet obesity. Indeed, the owners of obese dogs spend more than twice as long watching them eat^(^^16^^)^. The perception of a satisfied dog, even when on a weight management regimen could have a beneficial psychological impact on owners certainly help to ensure that owners persist with a weight programme who has been asked to reduce their dog's food intake. However, although some overweight dogs were included, the present studies did not assess obese dogs. Furthermore, satiety effects were not assessed when feeding at a level equivalent to that used for controlled weight loss. Therefore, additional studies, using obese dogs undergoing weight loss, are now needed.

To be sure that the satiety effect was due to volume and not macronutrient profile, we compared the satiety effect of two diets differing in volume but not formula. This study demonstrated that energy intake decreased when volume increased independently of macronutrient profile, thus confirming that the satiety effect observed with the volume-expanded diet was due to incorporation of air into food rather than the high level in starch compared with standard expanded dry diets.

The main limitation of these preliminary studies was that the population used was small; dogs were maintained in a colony environment and, for two of the experiments, only dogs of a single breed were used. As a result, the findings may not be relevant to a larger population of pet dogs in their home environment. A further limitation was the studies were short in duration and, as a result, longer-term satiety effects were not assessed. Furthermore, while increasing diet volume did slow food intake, the effect was relatively minor overall. It is unclear as to what effect this difference would have in the voluntary food intake of pet dogs, with different owners, with variable feeding strategies. As a result, additional studies are now needed, which use a larger and more diverse population of (ideally pet) dogs, monitored over longer periods.

In conclusion, results from the present study indicate that incorporating air into food provides a strategy to reduce energy intake in dogs and, consequently, could be a useful strategy for weight management in pets. A prospective clinical trial is now required to determine the effect on satiety in obese dogs during a weight-loss programme.
